# Supplementary material for: Cortical state dynamics and selective attention define the spatial pattern of correlated variability in neocortex
Source: Nat Commun. 2022 Jan 10;13:44. doi: 10.1038/s41467-021-27724-4 (PMC8748999; doi:10.1038/s41467-021-27724-4)
Supplement: Supplementary file 3 — Description of Additional Supplementary Files [file 41467_2021_27724_MOESM3_ESM.pdf]

Description of Additional Supplementary Files

File Name: Supplementary Movie 1

Description: Simulated activity in the dynamical-system network model. Each pixel in the movie represents On (yellow) and Off (blue) phases within a single column. The full network consists of 256 256 interacting columns simulated on a two-dimensional grid (upper panel). Lower panel shows the activity within a sub-population of 51 51 columns (red square in the upper panel). Spatiotemporal population activity forms local clusters with the size of a few columns, which propagate laterally as local irregular waves. Due to stochasticity of dynamics, the activity clusters do not propagate coherently across the entire network, but travel only locally until they fade or merge with other clusters. The spatial scale of activity clusters defines the exponential decay constant of noise correlations with lateral distance, i.e. the correlation length.
