# Supplementary material for: Cortical state dynamics and selective attention define the spatial pattern of correlated variability in neocortex
Source: Nat Commun. 2022 Jan 10;13:44. doi: 10.1038/s41467-021-27724-4 (PMC8748999; doi:10.1038/s41467-021-27724-4)
Supplement: Supplementary file 5 — Reporting Summary [file 41467_2021_27724_MOESM5_ESM.pdf]

nature research

Corresponding author(s): Tatiana Engel

Last updated by author(s): Nov 9, 2021

Reporting Summary

Nature Research wishes to improve the reproducibility of the work that we publish. This form provides structure for consistency and transparency in reporting. For further information on Nature Research policies, see our Editorial Policies and the Editorial Policy Checklist.

Statistics

For all statistical analyses, confirm that the following items are present in the figure legend, table legend, main text, or Methods section.

n/a	Confirmed	
☐	☒	The exact sample size (n) for each experimental group/condition, given as a discrete number and unit of measurement
☐	☒	A statement on whether measurements were taken from distinct samples or whether the same sample was measured repeatedly
☐	☒	The statistical test(s) used AND whether they are one- or two-sided<br>*Only common tests should be described solely by name; describe more complex techniques in the Methods section.*
☐	☒	A description of all covariates tested
☐	☒	A description of any assumptions or corrections, such as tests of normality and adjustment for multiple comparisons
☐	☒	A full description of the statistical parameters including central tendency (e.g. means) or other basic estimates (e.g. regression coefficient) AND variation (e.g. standard deviation) or associated estimates of uncertainty (e.g. confidence intervals)
☐	☒	For null hypothesis testing, the test statistic (e.g. F, t, r) with confidence intervals, effect sizes, degrees of freedom and P value noted<br>*Give P values as exact values whenever suitable.*
☒	☐	For Bayesian analysis, information on the choice of priors and Markov chain Monte Carlo settings
☒	☐	For hierarchical and complex designs, identification of the appropriate level for tests and full reporting of outcomes
☐	☒	Estimates of effect sizes (e.g. Cohen's d, Pearson's r), indicating how they were calculated

Our web collection on statistics for biologists contains articles on many of the points above.

Software and code

Policy information about availability of computer code

Data collection	Data were recorded using the Omniplex system (Plexon).
Data analysis	Data was analyzed with a custom code written in Matlab 2018a.

For manuscripts utilizing custom algorithms or software that are central to the research but not yet described in published literature, software must be made available to editors and reviewers. We strongly encourage code deposition in a community repository (e.g. GitHub). See the Nature Research guidelines for submitting code & software for further information.

Data

Policy information about availability of data

All manuscripts must include a data availability statement. This statement should provide the following information, where applicable:

- Accession codes, unique identifiers, or web links for publicly available datasets
- A list of figures that have associated raw data
- A description of any restrictions on data availability

The behavioral and electrophysiological data analyzed in this study are available as downloadable files at https://doi.org/10.6084/m9.figshare.16934326.v3. Source data are provided with this paper.

Field-specific reporting

Please select the one below that is the best fit for your research. If you are not sure, read the appropriate sections before making your selection.

☒ Life sciences ☐ Behavioural & social sciences ☐ Ecological, evolutionary & environmental sciences

For a reference copy of the document with all sections, see nature.com/documents/nr-reporting-summary-flat.pdf

Life sciences study design

All studies must disclose on these points even when the disclosure is negative.

Sample size	Sample sizes are similar to those used in other primate studies (Steinmetz and Moore, Neuron, 83, 496–506, 2014; Nandy and Reynolds, Neuron, 93, 235–246, 2017). The number of trials in each session was determined by the animals' ability to perform the task. The number of simultaneously recorded neurons was determined by the characteristics of the multielectrode arrays. The number of subjects (2) is standard for the primate studies.
Data exclusions	No data were excluded.
Replication	All of analysis results were successfully replicated in two macaque monkeys.
Randomization	Participants were not allocated into groups. No subject randomization was implemented
Blinding	Blinding is not relevant. Two macaque monkeys were used. There were no differences between the subjects that would conceivably create biases.

Reporting for specific materials, systems and methods

We require information from authors about some types of materials, experimental systems and methods used in many studies. Here, indicate whether each material, system or method listed is relevant to your study. If you are not sure if a list item applies to your research, read the appropriate section before selecting a response.

Materials & experimental systems

n/a	Involved in the study	
☒	☐	Antibodies
☒	☐	Eukaryotic cell lines
☒	☐	Palaeontology and archaeology
☐	☒	Animals and other organisms
☒	☐	Human research participants
☒	☐	Clinical data
☒	☐	Dual use research of concern

Methods

n/a	Involved in the study	
☒	☐	ChIP-seq
☒	☐	Flow cytometry
☒	☐	MRI-based neuroimaging

Animals and other organisms

Policy information about studies involving animals; ARRIVE guidelines recommended for reporting animal research

Laboratory animals	Two male monkeys (Macaca mulatta, 8–12 kg, between 6 and 9 years of age) were used in these experiments.
Wild animals	Wild animals were not used in this study.
Field-collected samples	No field collected samples were used in this study.
Ethics oversight	All experimental procedures were in accordance with NIH Guide for the Care and Use of Laboratory Animals, the Society for Neuroscience Guidelines and Policies, and Stanford University Animal Care and Use Committee.

Note that full information on the approval of the study protocol must also be provided in the manuscript.
